# Complete appendiceal inversion with local high-grade intraepithelial neoplasia in an adult female: a case report

**DOI:** 10.1186/s12893-019-0632-3

**Published:** 2019-11-11

**Authors:** Xinlu Liu, Ge Liu, Yanfeng Liu, Hongsheng Zhou, Liyu Yu, Yun Xu, Xue Song, Jing Zhang

**Affiliations:** 1 1st Department of general surgery, First Hospital of Dalian Medical University, No. 193 Union Road, Dalian City, Liaoning Province People’s Republic of China; 2 Operating Room, First Hospital of Dalian Medical University, No. 193 Union Road, Dalian City, Liaoning Province People’s Republic of China

**Keywords:** Appendiceal inversion, Appendiceal neoplasia, Surgery

## Abstract

**Background:**

Appendiceal inversion with neoplasia in adults is an extremely rare event with a reported incidence of < 0.01%. Preoperative diagnosis is very important for surgical treatment; however, it is very difficult to be exact.

**Case presentation:**

The patient was a 60-year-old woman with complaints of intermittent abdominal pain. Computed tomography and colonoscopy revealed a cecal mass, which was diagnosed as a tubulovillous adenoma in the preoperative colonoscopic biopsy. At surgery, the appendix was found to be completely inverted into the cecum. The cecum was partially resected, and surgical pathology examination confirmed a tubulovillous adenoma of the appendix with local high-grade intraepithelial neoplasia.

**Conclusions:**

Although preoperative diagnosis of appendiceal inversion with neoplasia may be often difficult due to its non-specific symptoms, clinicians should consider this disease entity when they encounter an intraluminal protruding cecal mass without visualization of the normal appendix on CT and colonoscopy.

## Background

Appendiceal inversion in adults is a rare event, with a reported incidence of approximately 0.01% [1]. Although computed tomography (CT) and colonoscopy may detect the condition, most reported cases have been misdiagnosed as appendicitis or ileocecal tumor, with the true diagnosis made only during or after surgery [2, 3]. Neoplasia of the appendix is another distinctly rare condition, with a reported incidence of only 0.08% [4, 5]. The combination of both appendiceal neoplasia and inversion, however, is extremely rare. We report a case of complete appendiceal inversion in the presence of a local high-grade intraepithelial neoplasia in an adult female, which was managed in our hospital, and discuss the clinical features, preoperative and postoperative diagnosis, and surgical considerations.

## Case presentation

**Fig. 1:**
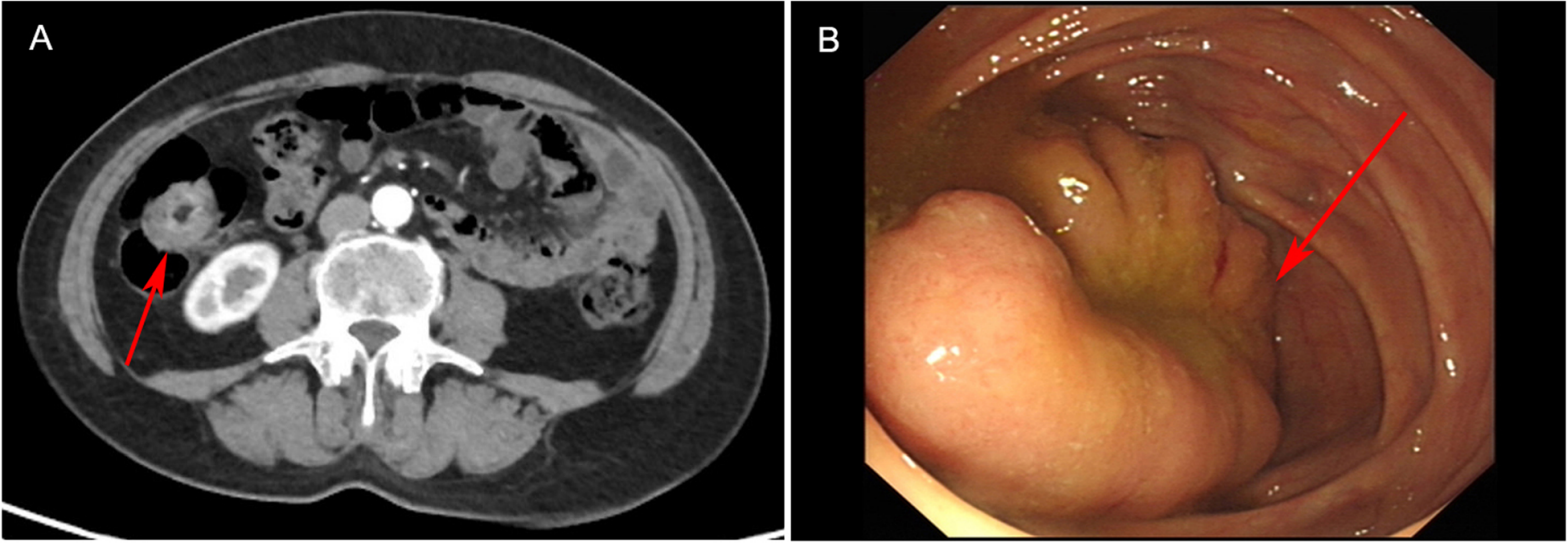
Preoperative findings. **a**. Computed tomography reveals a 3.5 × 2.5 cm intraluminal protruding mass with central fat component in the cecum. Regional lymph nodes are not enlarged. **b**. Colonoscopy reveals a long protruding lesion in the cecum, 3.5 cm in size

A 60-year-old woman was referred to the authors’ hospital with a 30-year history of intermittent abdominal pain, sometimes with nausea and vomiting, and occasionally with fainting. The symptoms usually subsided after a few minutes. The patient had no relevant surgical history. On physical examination, she was afebrile, and the abdomen, including McBurney’s point, was non-tender. Laboratory investigations revealed a white blood cell count of 4.41 × 10^9^/L and a hemoglobin level of 10.6 g/dL (106 g/L); her stool was negative for occult blood. Abdominal CT revealed a long, annular mass in the proximal ileocecal portion of the ascending colon. The mass was hollow in the center, and there were no enlarged peripheral lymph nodes (Fig. 1a). The uterine adnexa were normal. Colonoscopy revealed a long protruding lesion in the ileocecum with a velvety to granular surface, exhibiting the “coiled spring” sign (Fig. 1b). Local biopsy indicated tubulovillous adenoma.

**Fig. 2:**
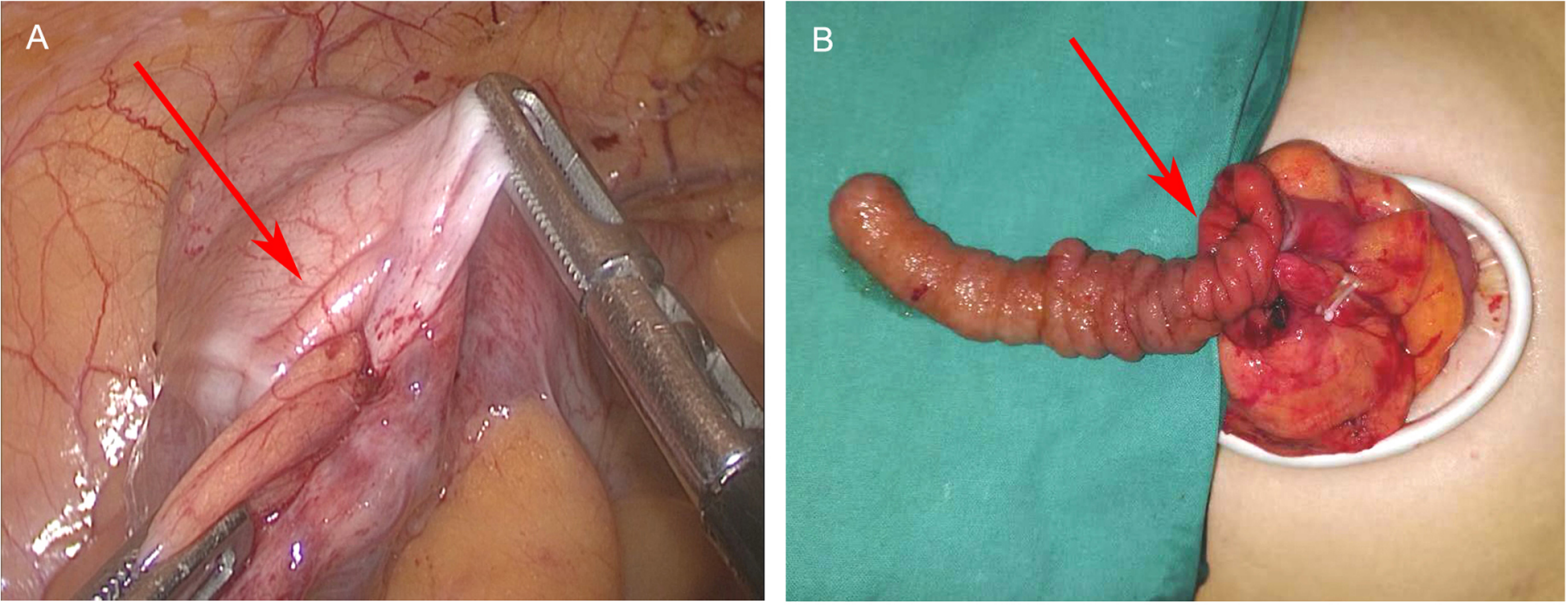
Intraoperative findings. **a**. On laparoscopic view, the appendix and its mesentery appear to be inverted into the cecum and cannot be flipped out. **b**. The inverted appendix with extroverted mucosa was pulled out of the cecum under direct visualization

On laparoscopy, the appendix was not visible at the end of the cecum; rather, the appendix and its mesentery were completely inverted into the cecum and could not be flipped out (Fig. 2a). No regional enlarged lymph nodes were found. Laparoscopic partial cecectomy was planned.

During the operation, after fully dissecting the mesenteries of the distal ileum and ascending colon, the cecum was lifted out and incised under direct vision. A mucosa-extroverted appendix was found completely inverted into the cecal cavity (Fig. 2b). The appendix and partial cecum were resected without lymph node dissection; The intraoperative frozen-section biopsy indicated that the surgical margin was tumor-free. The residual wall of the cecum was closed using a linear closure and reinforced with a figure-of-eight suture. The patient’s postoperative recovery was quick and uneventful, and she was discharged after 5 days. At the 10-month follow-up, there were no signs of complication or recurrence.

**Fig. 3:**
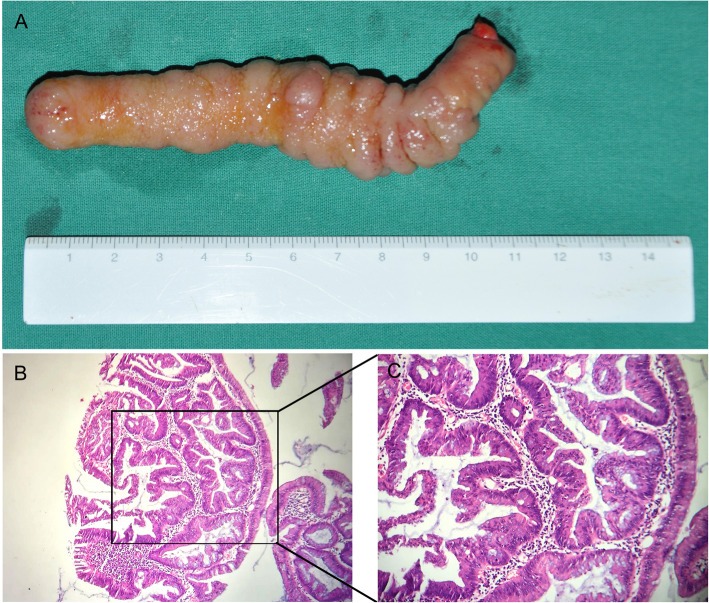
Postoperative findings. **a**. The appendiceal mucosa was completely everted, and the appendix measured 11 × 1.5 × 1.5 cm. The entire appendix mucosa is almost tubulovillous adenoma superficially. **b** and **c**. Microscopic examination of the resected specimen indicated a tubulovillous adenoma with local high-grade intraepithelial neoplasia (**b**: hematoxylin/eosin [H&E], original magnification × 100; **c**: H&E original magnification × 200)

On gross pathology, the appendix measured approximately 11 cm in length and 1.5 cm in external diameter. The extroverted mucosa was diffusely thickened and granular in appearance (Fig. 3a). Histopathology confirmed tubulovillous adenoma of the appendix with local high-grade intraepithelial neoplasia (Fig. 3b, c).

## Discussion and conclusion

Appendiceal inversion is a very rare condition, with an incidence of < 0.01% among surgical patients. It was first described by Mc Kidd in 1858 and, since then, there have been only a few cases reported in the literature [6]. Neoplasia of the appendix is another very rare entity, with an incidence of only 0.08% in resected cases [5], which in most cases arises from the carcinogenesis of premalignant lesion. The combination of both neoplasia and appendiceal inversion is regarded to be extremely rare.

Early and correct diagnosis of appendiceal inversion is important but difficult. First, patients usually experience no obvious symptoms. As in our case, the patient only experienced intermittent abdominal pain, accompanied by occasional nausea and vomiting. Second, there are no typical signs on ancillary examinations, and clinicians are likely to overlook it because of its rare incidence. In this case, CT revealed an occupying lesion in the cecum that mimicked an intracavitary tumor. Colonoscopy revealed a long polypoid mass, which was diagnosed on local biopsy as a tubulovillous adenoma.

McSwain categorized 5 types of appendiceal intussusceptions as follows: type I, the tip of the appendix is invaginated into the proximal appendix, which forms the intussusception; type II, the invagination starts at some point along the length of the appendix, and the intussusception is the appendiceal body; type III, the invagination starts at the junction of the appendix and cecum, and the intussusception is the cecum; type IV, the proximal appendix is invaginated into the distal appendix, which is the retrograde intussusception; and type V, the appendix is completely invaginated into the cecum. According McSwain’s classification of appendiceal intussusception, complete inversion of the appendix in the present case was type V [2, 7, 8].

Surgery is the primary mode of diagnosis and treatment for this type of disease. However, due to the lack of clear guidelines, the choice of surgical approach to appendiceal inversion remains controversial. Recommended surgical methods include appendectomy, partial cecal resection, ileocecal resection, and right hemicolectomy with peripheral lymph node dissection. The choice of surgical method depends mainly on the classification and estimation of the degree of malignancy of this disease by clinicians. Appendectomy, partial cecal resection, and ileocecal resection are usually considered in cases with low malignant potential. Right hemicolectomy or ileocecal resection with peripheral lymph node dissection should be performed when carcinoma is diagnosed preoperatively or during surgery. In the present case, only laparoscopic colectomy without lymph node dissection was performed based on the colonoscopic findings and nodal status, which is recommended in McSwain’s classification system [7]. The postoperative pathological examination of the resected specimen revealed a tubulovillous adenoma of the appendix with local high-grade intraepithelial neoplasia, which is a premalignant lesion proceeding to adenocarcinoma. Through our overall analysis of this case and its characteristics, we believe that local excision was appropriate.

In conclusion, appendiceal inversion is a very rare condition, especially when accompanied by premalignant lesion or carcinogenesis. Because of its non-specific clinical manifestations, most cases are misdiagnosed as appendicitis or cecal mass, which can lead to delayed or inappropriate treatment. Therefore, clinicians should devote a high degree of attention when they encounter an intraluminal protruding cecal mass without visualization of the normal appendix.

## Data Availability

As a case report, all data generated or analyzed are included in this article.

## Abbreviation

CT: Computed tomography
